# No-Touch Automated Room Disinfection after Autopsies of Exhumed Corpses

**DOI:** 10.3390/pathogens9080648

**Published:** 2020-08-12

**Authors:** Patryk Tarka, Aleksandra Borowska-Solonynko, Małgorzata Brzozowska, Aneta Nitsch-Osuch, Krzysztof Kanecki, Robert Kuthan, Barbara Garczewska

**Affiliations:** 1 Department of Social Medicine and Public Health; Medical University of Warsaw, 3 Oczki St., 02-007 Warsaw, Poland; anitsch@wum.edu.pl (A.N.-O.); kanecki@mp.pl (K.K.); 2 Department of Forensic Medicine, Medical University of Warsaw, 1 Oczki st., 02-007 Warsaw, Poland; borowska.solonynko@gmail.com (A.B.-S.); malgorzata.brzozowska@wum.edu.pl (M.B.); 3 Chair and Department of Medical Microbiology, Medical University of Warsaw, 5 Chalubinski st., 02-004 Warsaw, Poland; rkuthan@yahoo.com; 4 Laboratory of Mycology, Institute of Tuberculosis and Lung Diseases, 26 Płocka st., 01-138 Warsaw, Poland; b.garczewska@igichp.edu.pl

**Keywords:** forensic autopsy, exhumed body, no-touch room disinfection

## Abstract

Autopsies of exhumed bodies pose a risk of infections with environmental bacteria or fungi, which may be life-threatening. Thus, it is important to use effective methods of disinfection in forensic pathology facilities. In this study, we investigated the effectiveness of no-touch automated disinfection (NTD) system after autopsies of exhumed bodies. Directly after 11 autopsies of exhumed bodies, we used an NTD system based on a peroxone vapor to disinfect the air and surfaces. We measured microbial burden in the air and on surfaces before and after NTD. The NTD system reduced the mean bacterial burden in the air from 171 colony forming units (CFU)/m^3^ to 3CFU/m^3^. The mean fungal burden in the air decreased from 221 CFU/m^3^ to 9CFU/m^3^. The mean all-surface microbial burden was 79 CFU/100 cm^2^ after all autopsies, and it decreased to 2 CFU/100 cm^2^ after NTD. In conclusion, the peroxone-based NTD system was effective for decontamination of the air and surfaces in a dissecting room after autopsies of exhumed bodies.

## 1. Introduction

Forensic pathologists are at an increased risk of various infections, particularly those associated with hepatotropic viruses, coronaviruses, meningococci, or *Mycobacterium tuberculosis* [1,2,3,4,5]. Moreover, autopsies of exhumed bodies pose a particular risk of infections with pathogenic strains of bacteria or fungi from decomposing bodies or environment, which can be life-threatening even in immunocompetent people [5,6]. For example, *Aspergillus flavus*, often found in corpses, may cause fatal invasive aspergillosis [6,7]. A community of bacteria, fungi or other organisms colonizing the cadaver is called necrobiome [8]. Necrobiome may contain *Eurotium repens,* isolated from the surfaces of skin and bones of corpses [9] and molds such as *Penicillium oxalicum* and *Cladosporium colocasiae*, isolated from corpses preserved in low percentages (4%) of formaldehyde [10]. Fungi metabolize organic matter in situ and change both its biochemical and physicochemical properties and microbial community structure [11]. Pathogens may be acquired by inhalation (droplet, air dust, aerosol generated during the opening body), ingestion, direct skin contact or contact with infected surfaces in the dissection room (entry though pre-existing breaks in the skin, and through the mucous membranes of the eyes, nose or mouth) [12]. Thus, it is important to use effective methods of disinfection in forensic pathology facilities.

Historically, one of the agents used for disinfection of hospital rooms, laboratories, and sectional rooms was formaldehyde. However, toxic issues associated with formaldehyde led to the implementation of other, much safer gaseous substances. The group of methods based on advanced oxidation technologies/processes (AOT/AOP) seem to be particularly promising. These methods include ozonolysis in the presence of UV light (O_3_/UV); hydrogen peroxide and ozone (O_3_/H_2_O_2_), photocatalytic oxidation with the presence of titanium dioxide (TiO_2_) and Fenton system oxidation (H_2_O_2_/Fe^2+^). The common feature of these methods is the formation of peroxone, a highly biologically active compound [13,14]. Peroxone is formed as a result of redox reaction where the oxidative reagent is oxygen and/or its active forms such as ozone, H_2_O_2_, and peroxide radicals. In these reactions, the free radical mechanism is dominant, and the most important product is hydroxyl radical ^•^HO having high redox potential (2.8 V, Table 1).

In healthcare, no-touch automated disinfection (NTD) systems are gaining increasing popularity in addition to standard surface disinfection [16,17,18,19]. Typically, NTD systems use substances such as hydrogen peroxide or chlorine dioxide to automatically disinfect whole rooms [20,21,22]. Advanced oxidation processes, such as the combination of hydrogen peroxide with ozone (peroxone), are also used [23]. NTD systems are effective against bacteria, viruses, and fungi, including *Aspergillus* spp., *Penicillium* spp., and *Fusarium* spp. [24]. However, available data regarding fungal infection after autopsies of exhumed bodies are limited while the performance of the NTD system after autopsies has not been investigated so far. In this study, we investigated the effectiveness of a peroxone-based NTD system in decontaminating dissecting rooms after autopsies of exhumed bodies. To the best of our knowledge, this is the first study addressing the effectiveness of peroxone-based NTD system in disinfection following autopsies of exhumed bodies.

## 2. Results

Table 2 shows all the species of bacteria and fungi identified in the study. These organisms were identified after autopsies using VITEK^®^2 automated system. All identified microorganisms belong to environmental bacteria and fungi. 

**Figure 1:**
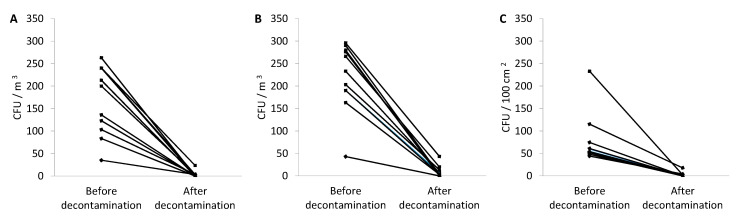
Bacterial burden in the air (**a**), the fungal burden in the air (**b**), and microbial burden on surfaces in dissecting room (**c**) following the autopsies of exhumed corpses before and after 1 h of no-touch decontamination.

After autopsies, the mean bacterial burden in the air was 171 (range 35–263) colony forming units (CFU)/m^3^, and it decreased to 3 (0–23) CFU/m^3^ after decontamination (Figure 1). The mean fungal burden in the air was 221 (43–290) CFU/m^3^, and it decreased to 9 (0–43) CFU/m^3^ after decontamination (Figure 1).

The mean all-surface microbial burden was 79 (44–238) CFU per 100 cm^2^ after all autopsies, and it decreased to 2 (0–18) CFU per 100 cm^2^ (Figure 1). Table 3 shows detailed contamination data for all surfaces together with respective safety levels. In 8 of 11 autopsies, the use of an NTD system resulted in no detectable microbiological contamination. In the three remaining autopsies, there was residual level 1 (two autopsies) and level 2 (one autopsy) contamination (Table 3).

## 3. Discussion

This study showed that peroxone-based NTD system was effective in decontaminating the air and surfaces in a dissecting room after autopsies of exhumed bodies. Importantly, the NTD system effectively reduced or eradicated both bacterial and fungal contamination. 

The literature addressing safety issues in dissection rooms recommend the use of personal protective equipment, such as gloves, goggles or masks during autopsies [2,3,4]. Current guidelines regarding decontamination are limited to cleaning and disinfection of surfaces and tools, depending on the potential infectious agent [26]. Recently, a European standard describing methods of disinfection of nonporous surfaces by automated distribution of chemicals was released [27]. This document will allow to apply a uniform standard to assess and compare the effectiveness of available NDT systems. 

NTD systems are used in so-called clean conditions, i.e., after initial cleaning and disinfection of surfaces. In our study, the level of microbiological contamination was not studied directly after initial cleaning with sodium hypochlorite. However, we found that the microbiological level in the air, where sodium hypochlorite was not used, decreased after the NTD system. The initial cleaning and disinfection followed by using NTD system should be considered as an integrated approach.

Decontamination of dissecting rooms is important to reduce the risk of infections, particularly after forensic autopsies of exhumed bodies, which can cause acute life-threatening infectious diseases. In our study, we identified several potentially pathogenic strains of bacteria and fungi after the autopsies (Table 2); however, the microbial burden was low. According to the EU regulations, the number of microorganisms in the air should not be greater than 500 CFU/m^3^ [25]. In our study, this threshold safety level was not reached after autopsies of exhumed bodies, even before NTD. Nevertheless, the NTD system reduced the number of microorganisms to even lower levels. Our results are in line with a previous study in which another NDS system based on hydrogen peroxide and silver cations was used to decontaminate hospital ventilation systems [28]. In that study, contamination with *Aspergillus fumigatus* was eradicated after disinfection. In our study, the microbial burden was not detectable after the use of NTD system in 8 of 11 autopsies, and in the remaining autopsies, the residual microbial contamination was low.

Fungal contamination is a particular concern after autopsies of exhumed bodies. The growth of fungi is often visible on exhumed bodies, and the spores of different fungi, such as *Aspergillus* spp. and *Penicillium* spp. are often present [5,29]. Fungi are very resistant to low humidity, and fungal conidia are able to survive for several decades even in liquid nitrogen or when lyophilized [30]. Fungi and mycotoxins may cause many diseases. For example, *Aspergillus flavus* is the etiological factor of sinusitis, keratitis, or skin lesions [31], and *Aspergillus flavus* or *Aspergillus fumigatus* may cause asthma or allergic pneumonitis [32,33,34,35]. Moreover, *Aspergillus* spp. may lead to life-threatening conditions, such as fatal invasive aspergillosis [6,7]. In our study, both *Aspergillus flavus* and *Aspergillus fumigatus* were identified. This is in line with findings of Schwarz et al., who also reported the presence of *Aspergillus fumigatus* on decomposed bodies [5]. 

Similarly to reports by Schwarz et al. [5] and Łukaszuk et al. [6], most fungi identified in our study were recognized as safe (belonging to risk group 1). Two fungal isolates reported in our study, Cladosporium spp. and Penicillium spp., are common environmental saprophytes. Nevertheless, epidemiological studies have shown an association between exposure to *Penicillium* and increased risk of wheeze, persistent cough, and higher asthma severity score [36]. Moreover, some species (classified as risk group ≤3) of *Cladosporium* spp. and *Penicillium* spp., may pose a risk of severe disease [37,38]. Some species previously classified as *Cladosporium* and *Penicillium* have been re-classified as risk group 3 pathogens, e.g., *Cladophialophora bantiana*, causing severe infections of central nervous system characterized by high mortality rate [39], and *Talaromyces (Penicillium) marneffei*, causing severe deep infections. Despite the fact that these species are mostly common in Thailand, Cambodia, Taiwan, and India, the cases of infections with these pathogens have also been reported in other regions [40]. The presence of *Cladosporium* spp. and *Penicillium* spp. on human corpses had been shown previously [41,42,43,44]. In contrast to other studies, we did not detect fungi belonging to *Candida* species, which are recognized as possibly allergenic [5,44].

This study has some limitations. Firstly, it was limited to a single dissecting room, and our observations need to be confirmed in other facilities. We used sodium hypochlorite for pre-cleaning and the residual chlorine could affect the microbial load at the time of assessment. The level of microbiological contamination was not studied directly after this procedure. Moreover, we used only one device for NTD, and other systems available on the market would require similar investigations. Nevertheless, because there is limited data on the use of NTD systems in dissecting rooms, our study presents useful information for facilities that conduct autopsies, particularly forensic ones.

In conclusion, NTD system seems promising for the decontamination of dissecting rooms after autopsies of exhumed bodies. Standards for the use of NTD systems in dissecting rooms are yet to appear, and until then the instructions delivered by manufacturers of specific devices should be followed. 

## 4. Materials and Methods

### 4.1. Study Design

We carried out 11 autopsies of exhumed bodies. Deaths occurred in 2010 as a result of injury; exhumations and autopsies were performed in 2018. We used the NTD system directly after autopsies of exhumed bodies. Before NTD was applied, the dissecting room was washed with sodium hypochlorite, according to the manufacturer’s instructions. Sodium hypochlorite is active against bacteria, viruses, spores, fungi, and mycotoxins [26]. The microbiological burden in the air and on various surfaces was measured directly after each autopsy and after decontamination with the Airdecon 200^TM^ system.

### 4.2. Device

We used the Airdecon 200^TM^ NTD system (Amity International, Barnsley, United Kingdom) in a dissecting room of a volume of 78 m^3^. The system sprays a peroxone vapor (a combination of hydrogen peroxide and ozone) to decontaminate all surfaces. The duration of decontamination was 1 h each time. Decontamination cycle consisted of the following phases: hydrogen peroxide phase, ozone phase followed by peroxone forming, and one-hour contact phase during which peroxone decomposed. For safety reasons, the device detects residual hydrogen peroxide level after the decontamination procedure. 

### 4.3. Microbiological Studies

We used the MicroBio MB 1 PLUS air sampler (Parrett, Bromley, United Kingdom) to measure the microbiological burden in the air. The MicroBio MB1 air sampler collects airborne micro-organisms on the surface of Petri dishes layered with the malt extract agar and the tryptic soy agar. After exposure, the dishes were removed and incubated under aerobic conditions, and the colony growths were counted. The count and the volume of air sampled were used to calculate the number of CFU per m^3^. In our study, we calculated the mean CFU/m^3^ values from three air samples. The device was placed 1.5 m from the floor, all doors and windows were closed, and 300 m^3^ of air was sampled thrice according to the PN—EN 13098 standard approved by the Polish Committee for Standardization [45].

We used 25 cm^2^, convex RODAC plates (Replicate Organism Detection and Counting) layered with tryptic soy agar with inactivators of inhibitory substances to measure contamination of surfaces (dissecting table, the working surface of the dissecting table, floor, working surface of tools trolley, sitting surface of chair). The plates were pressed (500 g/cm^2^) against the surface for 10 s, with no side movements, and were later incubated at 35 °C under aerobic conditions. The growth of microorganisms was measured in CFU per 100 cm^2^, and then expressed as a risk level according to the 1993 Draft European Standard CEN/TC 243/WG2 [46]; this was as follows: low risk, <10 CFU/100 cm^2^; moderate risk, 10–100; high risk, >100–1000, very high risk, >1000.

The identification of bacteria was carried out with an automatic detection system Vitek^®^2 (Biomerieux, Marcy-l’Étoile, France), according to the manufacturer’s instructions. VITEK^®^2 is an automated mass spectrometry microbial identification system that uses Matrix-Assisted Laser Desorption Ionization Time-of-Flight (MALDI-TOF) technology. Fungal species were identified by evaluation of their macroscopic and microscopic morphological features basing on the Atlas of Clinical Fungi [47].

## Figures and Tables

**Table 1:** Redox potential of the selected oxidizers [15].

Oxidative Agent	Redox Potential (V)
Hydroxyl radical ^•^HO	2.80
Molecular oxygen O_2_	2.42
Ozone in the acidic environment	2.07
Hydrogen peroxide in the acidic environment (H_2_O_2_/H)	1.78
Manganese ion (VII) in the acidic environment	1.69
Chloride dioxide	1.57
Chloride	1.36

**Table 2:** List of all organisms identified.

Bacteria	Fungi ^†^
*Bacillus simplex* *Bacillus vallismortis* *Enhydrobacter aerosaccus* *Gordonia sputi* *Kocuria rhizophila* *Kocuria rosea* *Kytococcus scedentarius* *Micrococcus luteus* *Moraxella osloensis* *Paenibacillus lantus* *Paenibacillus pabuli* *Paracoccus yeei* *Psychrobacter phenylpruvicus* *Staphylococcus auricularis* *Staphylococcus cohnii* spp. *Staphylococcus epidermidis* *Staphylococcus equorum* *Staphylococcus haemolyticus* *Staphylococcus hominis* *Staphylococcus pettekoferii* *Staphylococcus warneri* *Truicella otitidis*	*Alternaria* spp. (1) *Aspergillus flavus* (2) *Aspergillus fumigatus* (2) *Aspergillus niger* (1) *Chaetomium* spp. (1) *Cladosporium* spp. (≤3) *Fusarium* spp. (1) *Oidiodendron* spp. (1) *Penicillium citrinum* (1) *Penicillium* spp. (≤3) *Rhizopus* spp. (1) *Scopulariopsis* spp. (1) *Trichoderma* spp. (1)

^†^ risk group according to European Parliament Directive 2000/54/EC [25] shown in brackets.

**Table 3:** The microbiological burden on all surfaces before and after no-touch decontamination for all autopsies. Values represent microbiological contamination in CFU/100 cm^2^ (risk level) [25].

		Dissecting Table	The Working Surface of the Dissecting Table	Floor	The Working Surface of Tools Trolley	Sitting Surface of the Chair
Autopsy 1	Before	32 (2)	48 (2)	92 (2)	40 (2)	28 (2)
	After	0 (0)	0 (0)	0 (0)	0 (0)	0 (0)
Autopsy 2	Before	76 (2)	20 (2)	128 (3)	20 (2)	12 (2)
	After	0 (0)	0 (0)	0 (0)	0 (0)	0 (0)
Autopsy 3	Before	200 (3)	20 (2)	24 (2)	4 (1)	24 (2)
	After	0 (0)	0 (0)	0 (0)	0 (0)	0 (0)
Autopsy 4	Before	120 (3)	44 (2)	32 (2)	60 (2)	44 (2)
	After	0 (0)	0 (0)	0 (0)	0 (0)	0 (0)
Autopsy 5	Before	108 (3)	12 (2)	32 (2)	52 (2)	16 (2)
	After	8 (1)	4 (1)	0 (0)	0 (0)	8 (1)
Autopsy 6	Before	128 (3)	24 (2)	88 (2)	60 (2)	4 (1)
	After	0 (0)	0 (0)	0 (0)	0 (0)	0 (0)
Autopsy 7	Before	400 (3)	60 (2)	48 (2)	40 (2)	28 (2)
	After	0 (0)	24 (2)	25 (2)	24 (2)	16 (2)
Autopsy 8	Before	140 (3)	16 (2)	108 (3)	88 (2)	20 (2)
	After	0 (0)	0 (0)	0 (0)	0 (0)	0 (0)
Autopsy 9	Before	800 (3)	68 (2)	108 (3)	136 (3)	52 (2)
	After	4 (1)	4 (1)	4 (1)	0 (0)	0 (0)
Autopsy 10	Before	120 (3)	36 (2)	116 (3)	40 (2)	60
	After	0 (0)	0 (0)	0 (0)	0 (0)	0 (0)
Autopsy 11	Before	144 (3)	20 (2)	64 (2)	32 (2)	8 (1)
	After	0 (0)	0 (0)	0 (0)	0 (0)	0 (0)
